# Vaccination and Clinical Severity: Is the Effectiveness of Contact Tracing and Case Isolation Hampered by Past Vaccination?

**DOI:** 10.3390/ijerph10030816

**Published:** 2013-02-27

**Authors:** Kenji Mizumoto, Keisuke Ejima, Taro Yamamoto, Hiroshi Nishiura

**Affiliations:** 1 School of Public Health, The University of Hong Kong, Level 6, Core F, Cyberport 3, 100 Cyberport Road, Pokfulam, Hong Kong; E-Mails: mizumotokenji@gmail.com (K.M.); ejimakeisuke@gmail.com (K.E.); 2 Department of International Health, Nagasaki University Institute of Tropical Medicine and GCOE, 1-12-4 Sakamoto, Nagasaki 852-8523, Japan; E-Mail: y-taro@nagasaki-u.ac.jp; 3 Department of Mathematical Informatics, Graduate School of Information Science and Technology, The University of Tokyo, 7-3-1 Hongo, Bunkyo-ku, Tokyo 113-8656, Japan; 4 PRESTO, Japan Science and Technology Agency, 4-1-8 Honcho Kawaguchi, Saitama 332-0012, Japan

**Keywords:** symptom, immunization, epidemiology, mathematical model, contact tracing

## Abstract

While contact tracing and case isolation are considered as the first choice of interventions against a smallpox bioterrorist event, their effectiveness under vaccination is questioned, because not only susceptibility of host and infectiousness of case but also the risk of severe clinical manifestations among cases is known to be reduced by vaccine-induced immunity, thereby potentially delaying the diagnosis and increasing mobility among vaccinated cases. We employed a multi-type stochastic epidemic model, aiming to assess the feasibility of contact tracing and case isolation in a partially vaccinated population and identify data gaps. We computed four epidemiological outcome measures, *i.e.*, (i) the threshold of a major epidemic under the interventions; (ii) the expected total number of cases; (iii) the probability of extinction, and (iv) the expected duration of an outbreak, demonstrating that all of these outcomes critically depend on the clinical impact of past vaccination on the diagnosis and movement of vaccinated cases. We discuss that, even in the absence of smallpox in the present day, one should consider the way to empirically quantify the delay in case detection and an increase in the frequency of contacts among previously vaccinated cases compared to unvaccinated during the early stage of an epidemic so that the feasibility of contact tracing and case isolation in a vaccinated population can be explicitly assessed.

## 1. Introduction

Contact tracing and case isolation constitute a crucial element of non-pharmaceutical interventions against directly transmitted infectious diseases including smallpox [1,2,3]. Our society seeks for these countermeasures especially when we do not have any specific pharmaceutical interventions such as antiviral treatment and vaccination (or when we do not have substantial stock of the pharmaceutical agents to cover a large fraction of susceptible individuals in a population). Because of non-specific nature of the interventions, the contact tracing and case isolation have tended to be discussed assuming the absence of vaccination in public health contingency plans.

In the meantime, epidemiological study design and analysis of vaccine effects have greatly progressed during the past decades [4]. In addition to classifying epidemiological effects of vaccination into relative reductions in susceptibility and infectiousness, the reduced risks of symptomatic illness and severe clinical manifestations among vaccinated cases have been quantified using empirical datasets in clinical settings. Moreover, epidemiological impact of several different effects of vaccination on the reproduction number has been examined [5]. In the present day, one can theoretically predict the likely population impact of vaccination by employing a mathematical model, while realistically separating vaccine effects into various different types.

Because of independent progress of the abovementioned two subject areas, we have yet to understand how feasible the contact tracing and case isolation would be in the presence of vaccination in a population. This issue is motivated by previous epidemiological studies of smallpox [6,7] which estimated that previously vaccinated individuals by 1970s are probably not protected from contracting smallpox any longer in the present day. However, it was also implicated that the vaccinated individuals may still possess partially immunity against severe smallpox and death. The possession of partial protection should be good news as a whole for the prevention of smallpox epidemic in the absence of other interventions, but the residual immunity could induce unexpected adverse population effects. Namely, the residual immunity could mask signs and symptoms of vaccinated smallpox cases, thereby delaying the diagnosis or detection of cases, and moreover, the vaccinated cases may be more mobile than unvaccinated ones due to mild clinical symptoms [6].

We would like to assess if the contact tracing and case isolation, the first choice of interventions against smallpox, can remain feasible in a partially immune population. The present study is aimed to describe the transmission dynamics that involves contact tracing and case isolation under vaccination using a mathematical model, assessing the feasibility and identifying associated data gaps.

## 2. Methods

### 2.1. Mathematical Model

We start with considering the transmission dynamics in a randomly mixing population, although a heterogeneous model will be considered later. Let *R*_0_ be the basic reproduction number of an infectious disease, representing the average number of secondary cases generated by a typical primary case throughout the course of its infectiousness in a fully susceptible population [8]. In a common transmission dynamics written by ordinary differential equations (e.g., the so-called “Susceptible-Infectious-Removed (SIR)” model), *R*_0_ may be decomposed as the product of the transmission rate *β* and the average duration of infectiousness 1/*γ*. Under contact tracing practice which is accompanied by case isolation, we assume that the contact tracing takes place before the traced exposed individuals become infectious and that a fraction *q* of the total contacts of primary case can be traced and are effectively prevented. The average number of secondary cases generated by a primary case under the contact tracing is thus (1 − *q*)*R*_0_.

We describe a population in which only a part of them are fully protected from infection due to vaccination that took place in advance of an epidemic. Due to the vaccination practice, a fraction *v*_f_ is assumed as fully immune, while a fraction *v*_p_ is partially immune (e.g., not protected from smallpox but protected from the severe illness). Partially immune individuals are assumed to have a reduced susceptibility by a factor *α*_s_ ≤ 1. Even provided that the partially immune individuals are infected, their infectiousness is assumed to be *α*_i_ times that among fully susceptible individuals (where *α*_i_ ≤ 1). The relative reductions in susceptibility and infectiousness are common assumptions [4]. Moreover, we assume that vaccination elicits protection from severe disease. Because of the reduced severity, cases among partially immune individuals would be more mobile and have a greater contact rate than cases arising from susceptible individuals by a factor *α*_m_ ≥ 1. Lastly, the reduced severity may not only improve clinical outcomes but also delay diagnosis, and thus, we assume that the duration of infectious contact is lengthened by *α*_d_ times (where *α*_d_ ≥ 1). It should be noted that the last two effects, *i.e.*, *α*_m_ and *α*_d_, are vaccine-induced modifications in behavior, and thus, the estimates could vary with calendar time, geographic location and during the course of an epidemic (see Discussion). For simplicity and for the exposition of our modeling results, we focus on the early stage of an epidemic in a single hypothetical setting. In summary, we consider four individual effects of vaccination among which two act as protective (*i.e.*, reduce the average number of secondary cases per single primary case), while the remaining two factors, *α*_m_ and *α*_d_, can increase the reproduction number of partially immune individuals relative to fully susceptible individuals.

Under the abovementioned scenario, we assess four epidemiological outcome measures, including: (i) the threshold of a major epidemic under the interventions (contact tracing/case isolation and vaccination); (ii) the probability of extinction given a single vaccinated or unvaccinated index case; (iii) the expected number of cases throughout the course of an epidemic, and (iv) the expected duration of a minor outbreak.

To describe the transmission dynamics under vaccination, we employ the next-generation matrix. We split the population into two sub-groups by vaccination history and the average number of secondary cases in sub-group *i* generated by a single primary case of sub-group *j* is denoted by *R*_ij_ (where the subscripts *i* and *j* represent the vaccination history in which vaccinated individuals are denoted by 1 and otherwise 0). The average numbers of within- and between-group transmissions that are seen among those who are capable of causing further cases are parameterized as follows:

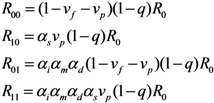
(1)

Namely, among unvaccinated contacts caused by unvaccinated primary cases, the proportion of those who do not possess residual immunity (1 − *v*_f_ − *v*_p_) and contact tracing of primary cases (1 − *q*) are multiplied to *R*_0_. When the contacts are vaccinated, partially protected population *v*_p_ has a reduced susceptibility by *α*_s_ times as compared to unvaccinated. When the primary case is vaccinated, the transmissibility is multiplied by *α*_i_*α*_m_*α*_d_ due to individual effects of vaccination on the primary case as mentioned above. Although the reproduction numbers (1) are heuristically described, *R*_ij_ similar to Equation (1) can be derived from a variety of equation systems that adopt the abovementioned assumptions. The next-generation matrix, **K**, is written as:

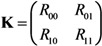
(2)

The effective reproduction number, *R*_v_, of a population with vaccine-induced immunity is defined as the dominant eigenvalue of the next-generation matrix:

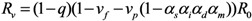
(3)

For the sake of clarity in our theoretical exposition (to apply model-based findings to the practical issue of smallpox control), we focus on the spread of infection that can be expected to be contained in the absence of partial immunity. Considering that 30% or more of the present day population has never been vaccinated, the assumption of “successful containment” indicates that the contact tracing is highly effective in preventing secondary transmission [1] (e.g., the initial attack size should not be too large to contain smallpox by means of contact tracing and case isolation). Namely, *R*_v_ < 1 for *v*_p_ = 0, or equivalently, (1 − *q*)(1 − *v*_f_)*R*_0_ < 1. 

### 2.2. Epidemic Threshold and Vaccine Effects

Let *n*_u,i_ and *n*_v,i_ be the numbers of unvaccinated and vaccinated cases in generation *i*, respectively. For mathematical convenience, here we focus on the exponential growth (linear phase) alone. Given the initial numbers of *n*_u,0_ unvaccinated and *n*_v,0_ vaccinated index cases, the *i*-th generation is written as:

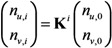
(4)

The total number of unvaccinated and vaccinated cases, *N*_u_ and *N*_v_, throughout the course of an epidemic is calculated as:

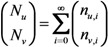
(5)

Combining Equations (4) and (5), we get:

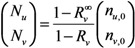
(6)
which results from geometric series and converges only in the case of *R*_v_ < 1. A major epidemic, which does not decline to extinction without substantial depletion of susceptible individuals or concerted effort of control, occurs if and only if *R*_v_ > 1. Solving the inequality with respect to vaccine effects, we obtain the following condition that allows a major epidemic to occur:

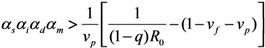
(7)

We assume that an outbreak starts with a single index case. In the sub-critical case (*i.e.*, *R*_v_ ≤ 1), the total number of cases converges to:

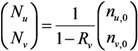
(8)

If an epidemic starts with a single infected individual who experiences infection at random, the probability that the index case is vaccinated is given by *v*_p_*α*_s_/(1 − *v*_f_ − *v*_p_ + *v*_p_*α*_s_). Otherwise, he/she is unvaccinated with the probability (1 − *v*_f_ − *v*_p_)/(1 − *v*_f_ − *v*_p_ + *v*_p_*α*_s_). Using this initial condition, the total number of cases who are capable of causing secondary transmissions is:

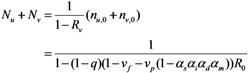
(9)

It should be noted that *N*_u_ + *N*_v_ increases if:

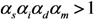
(10)

Caution must be exercised with respect to the total number of cases that is different from the calculation in Equation (9), because the abovementioned model has omitted the cases who were traced perfectly and isolated before developing infectiousness. That is, in addition to *R*_00_ + *R*_10_ in Equation (1), each unvaccinated cases have actually infected additional (1 − *v*_f_ − *v*_p_ + *v*_p_*α*_s_)*qR*_0_ cases who were perfectly traced and were not involved in further transmission dynamics given their own infections. Similarly, in addition to *R*_01_ + *R*_11_ in Equation (1), each vaccinated cases have caused *α*_i_*α*_m_*α*_d_(1 − *v*_f_ − *v*_p_ + *v*_p_*α*_s_)*qR*_0_ cases who were traced. Thus, the total number of cases *Z* is written as:

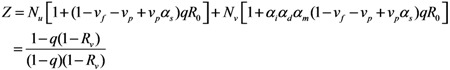
(11)

### 2.3. Probability of Extinction and Vaccine Effects

Whereas we consider a special case *R*_v_ < 1 for *v*_p_ = 0, a combined vaccine effect that satisfies in Equation (7) for *v*_p_ ≥ 0 can lead to a major epidemic. We thus consider the relationship between the probability of extinction and vaccine effects in the following by continuing to employ the abovementioned multi-type branching process approximation.

As adopted above, we label unvaccinated as type 0 and vaccinated as type 1. To ease the computation of the probability of extinction, we assume that the infectious period is exponentially distributed so that the multi-type branching process model can be rewritten as a multivariate birth-and-death process [9,10,11,12]. Let *γ*_i_ (*i* = 0 or 1) be the recovery rate of infectious individuals of type *i* and *β*_ij_ (0 ≤ *i*, *j* ≤ 1) be the rate of increase in infectious individuals (*i.e.*, the so-called “birth rate” of birth-and-death process) of type *i* generated by a primary case of type *j*. Considering a large population that consists of fully susceptible individuals, each element of the next-generation matrix is written as *R*_ij_ = *β*_ij_/*γ*_j_. 

Let *F* be the probability generating function of the multi-type branching process, *i.e.*,

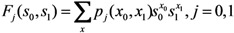
(12)
where *p*_j_(**x**) is the probability that an individual of type *j* causes the *x*_0_ unvaccinated and *x*_1_ vaccinated cases in the next generation. Following our foregoing study [9], we have *F*_j_(**s**) with an exponentially distributed infectious period that permits us to rewrite Equation (12) as:

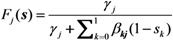
(13)
for *j* = 0 or 1. As we have already discussed elsewhere [11,12], Equation (13) is simplified to:

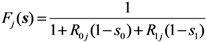
(14)

Any introduction of initial cases decline to extinction with probability 1 as long as *R*_v_ ≤ 1. In the case of *R*_v_ > 1, the extinction probability by generation *t*, ***π*^t^(*s*)** is described by using that at an earlier generation *t* − 1, *i.e.*,

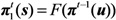
(15)
where ***u*** represents the initial condition, (*n*_u,0_, *n*_v,0_) = (0,1) or (1,0). Since we consider a two-host population (*i.e.*, unvaccinated and vaccinated cases), the probabilities of extinction at generation *t* given a single unvaccinated, ***π*^t^**(0,1) is given as a solution of the following simultaneous equations:

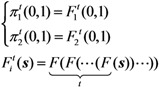
(16)

The probability of extinction, ***π*** can be obtained by taking the limit of *t*, which corresponding to:

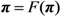
(17)
or equivalently:

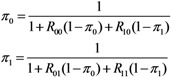
(18)
where *π*_0_ and *π*_1_ are the probabilities of extinction given one unvaccinated and vaccinated case, respectively. As practiced with many other branching process models, each of the secondary cases of type *i* generated by a primary case becomes an ancestor of an independent sub-processes (which restarts with a type *i* individual) behaving identically among the same type *i* [13]. Because of this multiplicative nature, the probability of extinction given multiple index cases (*a*_0_, *a*_1_) is calculated as:

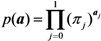
(19)

The expected duration of an epidemic, E(*T*) is calculated as:

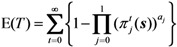
(20)


Since the abovementioned descriptions rest on homogeneous mixing assumption between vaccinated and unvaccinated individuals, we additionally consider a heterogeneous pattern of transmission, at least by assuming that there are more frequent contacts within unvaccinated subpopulation as compared to between vaccinated and unvaccinated individuals. To address the so-called “assortative mixing” (*i.e.*, a type of mixing in which a substantial fraction of contacts are reserved for within-group mixing), here we adopt the preferred mixing assumption [14]. Let *θ* be the proportion of contacts that are spent for within-group mixing, the element of the next-generation matrix for the heterogeneously mixing population is parameterized as:

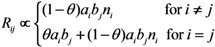
(21)
where *a*_i_ measures the susceptibility of sub-group *i*, *i.e.*,

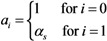
(22)


Similarly, *b*_j_ measures the infectiousness of primary cases in sub-group *j*, *i.e.*,

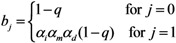
(23)
*n*_i_ scales the population size of sub-group *i*:

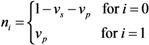
(24)


Note that the parameterization in Equation (21) would result in (1) if *θ* = 0 (*i.e.*, random mixing). When *θ* = 1, all transmission occurs within an identical group, and this type of transmission is referred to as fully assortative.

### 2.4. Numerical Illustration

We consider a hypothetical scenario of biological warfare using variola virus. In advance of the bioterrorist event, we assume that no one possess full protection against smallpox any longer, and thus, *v*_f_ = 0 [6,15]. On the other hand, we assume that *v*_p_ = 0.3 or 30% of the population still possesses partial protection (nevertheless, it should be noted that the fraction of susceptible individuals would continue to increase as time goes by). As mentioned above, our scenario is supposed to be a subcritical process in the absence of partial protection [6]. Namely, *R*_v_ < 1 for *v*_p_ = 0. This leads to (1 − *q*)(1 − *v*_f_)*R*_0_ < 1 (or (1 − *q*)*R*_0_ < 1 due to *v*_f_ = 0). In the absence of intervention, *R*_0_ is crudely assumed to be 5 which is in line with the goal of vaccination coverage during the Intensified Smallpox Eradication Programme without accounting for other interventions [16] and also with the published estimate of 6.85 if accompanied by contact tracing [17,18]. The protective effect of contact tracing, *q* is arbitrarily assumed as 0.8 due to an assumption of sub-critical process (*i.e.*, to adopt an assumption of *R*_v_ < 1). One should remember that these arbitrarily allocated *R*_0_ and *q* are very influential in discussing the feasibility of containment (using Equation (3)). Under these assumptions, we vary the combined effect of vaccination, denoted by *α*_s_*α*_i_*α*_m_*α*_d_, while we adopt a fixed value of *α*_s_ at 0.8 given that a historical household data with probably limited vaccine potency indicates that susceptibility is reduced by a factor of 0.69 [16]. Varying the combined effect of vaccination from 0 to 2, we calculate the estimate of the effective reproduction number, the expected total number of cases, the probability of extinction, and the expected duration of an outbreak. All statistical data were analyzed using a statistical software JMP version 9.0.0 (SAS Institute Inc., Cary, NC, USA).

## 3. Results

### 3.1. Epidemic Threshold

**Figure 1:**
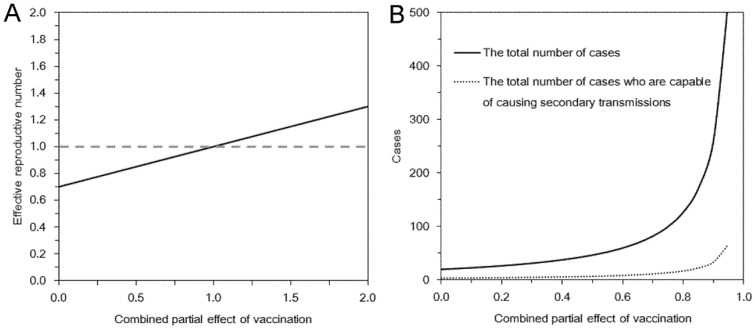
Epidemic threshold and the total number of cases under vaccination and contact tracing. In both panels, the horizontal axis represents the product *α*_s_*α*_i_*α*_m_*α*_d_ that measures the partial effects of vaccination. (**A**) The effective reproduction number under vaccination and contact tracing. (**B**) The expected total number of cases and the total number of cases who are capable of causing secondary transmissions. The basic reproduction number was assumed as 5. A fraction *q* = 0.8 of contacts was assumed to be protected by contact tracing. Vaccination was assumed to have conferred no full protection but partial protection among 30% of the population. Among partially protected individuals, susceptibility was assumed to be reduced by a factor of 0.8.

The effective reproduction number, *R*_v_, under vaccination and contact tracing/case isolation is computed using the assumed numerical values for smallpox and varying the uncertain product, *α*_s_*α*_i_*α*_m_*α*_d_, of combined effect of vaccination from 0 to 2 (Figure 1(A)). As can be seen from Equation (3), the reproduction number is a linear function of the combined vaccine effect. Also, the reproduction number leads to be supercritical (*i.e.*, *R*_v_ > 1) as long as the inequality in Equation (7) is not satisfied. Figure 1(B) shows the total number of cases based on Equations (9) and (11). Due to the use of geometric series 1/(1 − *R*_v_) in the expectations, the expected number of cases dramatically increases as *α*_s_*α*_i_*α*_m_*α*_d_ becomes close to 1. When *α*_s_*α*_i_*α*_m_*α*_d_ is greater than 1 in our hypothetical setting and Equation (7) is met, we would have *R*_v_ > 1 and a major epidemic could occur. As long as the product *α*_s_*α*_i_*α*_m_*α*_d_ is less than 0.9, the total number of cases is kept below 300, indicating the critical importance in quantitatively measuring *α*_s_*α*_i_*α*_m_*α*_d_.

### 3.2. Multi-Type Branching Process

**Figure 2:**
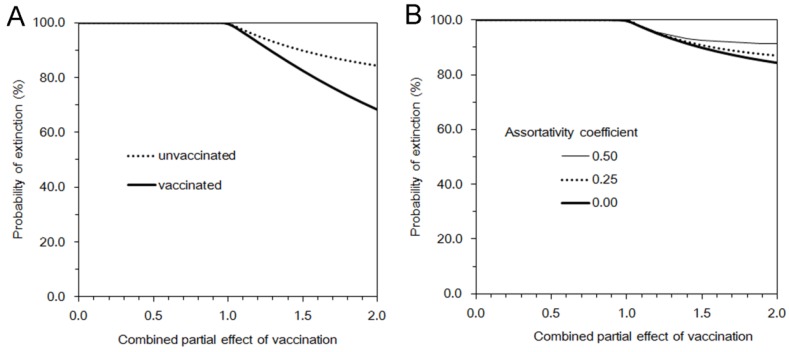
Probability of extinction and combined effect of vaccination. In both panels, the horizontal axis represents the product *α*_s_*α*_i_*α*_m_*α*_d_ that measures the partial effects of vaccination. (**A**) Probability of extinction given a single infected individual is compared by vaccination history of the index case. Random mixing assumption was adopted. (**B**) Probability of extinction given a single unvaccinated index case with different assortativity coefficient values.

The probability of extinction is examined in Figure 2(A) as a function of the combined effect of vaccination. As long as *α*_s_*α*_i_*α*_m_*α*_d_ satisfies inequality in Equation (7), we have *R*_v_ ≤ 1, and thus, the probability of extinction given a single infected individual is always 1. Otherwise, the extinction probability lies between 0 and 1. Under the examined scenario with the relationship *R*_00_ + *R*_10_ < *R*_01_ + *R*_11_ for *α*_i_*α*_m_*α*_d_ > 1, an introduction of single vaccinated individual is more risky to cause an epidemic than introducing an unvaccinated case into the population. For instance, when *α*_s_*α*_i_*α*_m_*α*_d_ = 3, the probabilities of extinction given an unvaccinated and a vaccinated case are 78.8% and 49.8%, respectively. We also examined the impact of assortative (heterogeneous) mixing on the probability of extinction given a single unvaccinated index case (Figure 2(B)). As can be intuitively expected, the greater the assortativity coefficient is, the greater the probability of extinction would be. 

**Figure 3:**
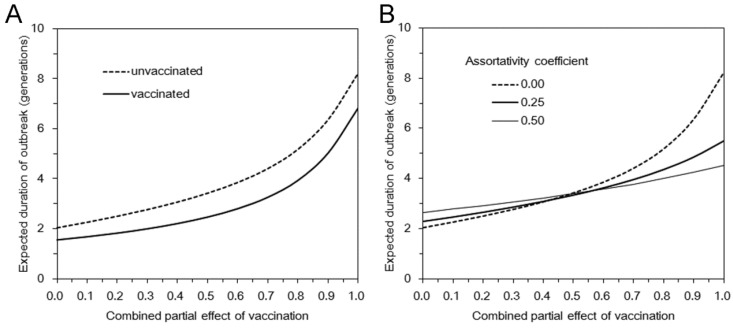
Expected duration of minor outbreak under vaccination. In both panels, the horizontal axis represents the product *α*_s_*α*_i_*α*_m_*α*_d_ that measures the partial effects of vaccination. (**A**) Expected duration of outbreak given a single infected individual is compared by vaccination history of the index case. Random mixing assumption was adopted. (**B**) Expected duration of outbreak given a single unvaccinated index case with different values of assortativity coefficient.

Figure 3(A) shows the expected duration of outbreak for a subcritical process which is examined as a function of the combine effect of vaccination. Since we consider the subcritical process with *R*_v_ ≤ 1 (*i.e.*, there would be only minor outbreaks) or *α*_s_*α*_i_*α*_m_*α*_d_ ≤ 1, introducing an unvaccinated individual as index case would yield a longer duration of an outbreak as compared to introducing a vaccinated case. The expected duration of minor outbreak within the assumed parameter space was overall shorter than 10 generations (*i.e.*, less than 150 days) and was not dramatically extended even when *R*_v_ becomes closer to a critical level. The impact of heterogeneous mixing on the duration of an outbreak given an unvaccinated index case is examined in Figure 3(B). 

For each combined effect of vaccination, the effective reproduction number with different assortativity coefficient was kept identical to allow comparisons. The basic reproduction number was assumed as 5. A fraction *q* = 0.8 of contacts was assumed to be protected by contact tracing. Vaccination was assumed to have conferred no full protection but partial protection among 30% of the population. Among partially protected individuals, susceptibility was assumed to be reduced by a factor of 0.8. Note that vertical axis of Figure 3 is expressed in generations of infection (the mean per generation is about 15 days).

Since an unvaccinated case is introduced, lower assortativity leads to observe longer durations of outbreak for *α*_s_*α*_i_*α*_m_*α*_d_ > 0.5. However, the advantage of low assortativity is diminished as *α*_s_*α*_i_*α*_m_*α*_d_ becomes smaller (*i.e.*, as the combined vaccine effect becomes larger).

## 4. Discussion

The present study modeled the transmission dynamics of an infectious disease, considering smallpox for the exposition and examining the contact tracing and case isolation in a partially immune population. Four different vaccine effects were considered, and especially, we took into account two specific effects that would adversely work for the epidemic control, *i.e.*, the delay in detecting cases and increase in mobility among vaccinated cases. In some unfavorable scenarios, we have shown that the negative impact of vaccination on disease control can lead to observing worse epidemic outcomes than the contact tracing and case isolation in the absence of vaccination. Thus, using a linear model written as a multi-type branching process approximation, the threshold of a major epidemic, the total number of cases, the probability of extinction and the duration of an outbreak were computed as a function of the total vaccine effects. We have demonstrated that all four epidemiological outcomes are greatly influenced by the assumed values of the combined effect of vaccination, which could partly lead to questioning the feasibility of successful containment of smallpox by means of contact tracing and case isolation. Nevertheless, such vaccination effects have yet to be empirically estimated.

Our study is the first to identify the associated data gap that would influence the feasibility of implementing contact tracing and case isolation in the event of a bioterrorist attack or any other opportunities (e.g., containment phase of a pandemic influenza). Among four different effects, the delay in detecting cases and an increase in the frequency of movement are two critical variables that must be measured urgently and compared between vaccinated and unvaccinated cases. Nevertheless, at a clinical setting, the relative difficulty in clinical diagnosis of vaccinated cases as compared with unvaccinated has not been routinely measured as one of biological effects of vaccination. Moreover, although there have been published studies that showed the reduced frequency of contact during symptomatic period (as compared with during the incubation period) [19], the frequency of contact (or the frequency of physical movement) has not been compared between vaccinated and unvaccinated cases. More importantly, two effects, *i.e.*, *α*_m_ and *α*_d_, are associated with behavioral aspect, and thus, it is likely that the estimates would not be regarded as biological constants, and rather, could vary with time and space. The estimates can also greatly vary with the recognition of a bioterrorist attack during the course of an epidemic. Although we have focused on the early stage of an epidemic in a single hypothetical setting and fixed these parameters as if these were constants, such assumptions may be subject to explicit evaluation.

To quantitatively measure these two effects (by assuming that these two are constants during the early stage of an epidemic), there would be two practical difficulties in the empirical observations. First, we do not have a widely accepted scale in measuring both vaccine effects in the way that directly influences the transmission dynamics (or the next-generation matrix). Second, smallpox has already been eradicated and our society does not have an opportunity to conduct any further empirical observations of naturally infected individuals. As a potential solution, one could consider gathering expert opinion [20] among those who experienced smallpox control or clinical management of smallpox cases. In fact, the effectiveness of post-exposure vaccination against smallpox has been explored based on a Delphi survey among experts [21]. Another possible solution is to use video or other pictorial materials that permit medical experts to judge the diagnoses of patients under hypothetical scenarios. Even a quick clinical diagnosis using photographs of smallpox patients is useful for training purpose among physicians in the present day (and such photographs can act as a precious learning material). In such an instance, the success probability of correct diagnosis of smallpox in vaccinated individuals (e.g., as compared to unvaccinated cases) may be used as a complimentary parameter that describes the vaccine-induced delay in diagnosis. On the other hand, we missed opportunities to directly measure the reduced mobility of vaccinated smallpox cases in humans. As complimentary information, the differential mobility between vaccinated and unvaccinated cases of other similar viral diseases (e.g., measles, chickenpox and rubella) may be studied.

Three technical limitations must be noted briefly. First, our model has been kept simple, and the realism has remained small. Our framework can be extended to realistic scenarios including the dynamics on a heterogeneous network [22,23,24,25], and future modeling studies should address this point. Second, the above discussed vaccine effects have not been supported by empirical data. Moreover, other vaccine effects (*i.e.*, other than the delay in diagnosis and increased mobility) may well have been missed. Third, not only the combined vaccine effect, but also the actual immunity levels, *i.e.*, *v*_f_ and *v*_p_, remain unknown in many epidemiological settings. Without understanding the background immunity levels, it is difficult to directly quantify the epidemic threshold using empirical data [26].

Although we have multiple tasks to be completed, we believe that the present study has contributed much to literature by identifying the associated data gaps in exploring the feasibility of contact tracing and case isolation under vaccination practice [25,27]. Employing various empirical approaches, the epidemiological adverse effects of vaccination should be statistically estimated in the future.
